# Relapsing-Remitting Multiple Sclerosis diagnosis from cerebrospinal fluids via Fourier transform infrared spectroscopy coupled with multivariate analysis

**DOI:** 10.1038/s41598-018-19303-3

**Published:** 2018-01-18

**Authors:** Dilek Yonar, Levent Ocek, Bedile Irem Tiftikcioglu, Yasar Zorlu, Feride Severcan

**Affiliations:** 1 Department of Biological Sciences, Middle East Technical University, 06800 Ankara, Turkey; 2 Izmir Tepecik Education and Research Hospital, Neurology Clinic, 35180 Izmir, Turkey; 3 Biophysics Department, Faculty of Medicine, Altinbas University, 34147, Bakirkoy, Istanbul, Turkey

## Abstract

Multiple sclerosis (MS) is a chronic, progressive, inflammatory and degenerative disease of central nervous system. Here, we aimed to develop a method for differential diagnosis of Relapsing-Remitting MS (RRMS) and clinically isolated syndrome (CIS) patients, as well as to identify CIS patients who will progress to RRMS, from cerebrospinal fluid (CSF) by infrared (IR) spectroscopy and multivariate analysis. Spectral analyses demonstrated significant differences in the molecular contents, especially in the lipids and Z conformation of DNA of CSF from CIS, CIS to RRMS transformed (TCIS) and RRMS groups. These changes enables the discrimination of diseased groups and controls (individuals with no neurological disease) from each other using hierarchical cluster and principal component analysis. Some CIS samples were consistently clustered in RRMS class, which may indicate that these CIS patients potentially will transform to RRMS over time. Z-DNA band at 795 cm^−1^ that is existent only in diseased groups and significant increase in carbonyl amount, decrease in amideI/amide II and lipid/protein ratios observed only for RRMS groups can be used as diagnostic biomarkers. The results of the present study shed light on the early diagnosis of RRMS by IR spectroscopy complemented with multivariate analysis tools.

## Introduction

Multiple sclerosis (MS) affects approximately 2.5 million people worldwide, ranking as one of the most prevalent neurodegenerative disease, and is the cause of disability among young adults especially in Europe and North America^1^. MS is a chronic, slowly progressive, inflammatory, demyelinating and degenerative disease of the central nervous system (CNS), characterized by an autoimmune inflammation^2–5^. The pathogenesis of MS encloses the immune mediated degradation of myelin, which is a fatty sheath surrounding the nerve fibres of the CNS and the axons, and progressive neuronal degeneration. The exact etiology of the disease is still unclear, in part owing to a lack of sensitivity in current techniques used to detect the onset and progression of the disease at early time points^6,7^. The disease has substantial clinical heterogeneity, but most patients who continue to develop MS initially experience a single demyelinating event, referred as clinically isolated syndrome (CIS). Patients experiencing CIS do not reveal evidence of the dissemination in time or space that is required for the diagnosis of multiple sclerosis, but are under high risk of developing this disease^5,8^. The clinical course of MS may follow different progression patterns, and one of them is the relapsing-remitting MS (RRMS) pattern that affects 85% of the MS patients. During the course of RRMS, patients experience periods of neurological dysfunction, named as relapses, which is followed by partial or complete clinical improvement (remissions)^9^. RRMS commonly begins with a CIS characterized by an acute or subacute episode of neurological disturbance due to a single white matter lesion located in the optic nerve, brainstem or spinal cord^8,10^. Prospective studies demonstrates that 60–70% of CIS patients develop a second clinically evident demyelinating event within 20 years and will, therefore, be diagnosed with clinically definite MS (CDMS)^11,12^. Clinical trials performed in CIS patients have shown that between 38% and 45% of untreated patients convert to CDMS within 2 years^5,13^. Therefore, it is important to differentiate the CIS and RRMS groups and also to identify CIS patients who will progress to RRMS.

Pathological conditions induce significant structural and functional alterations in biological systems. These alterations are directly reflected in the vibrational spectra of the studied samples and can be determined rapidly and sensitively without using any external agents by Fourier transform infrared (FTIR) spectroscopy^14,15^. Since the mid-IR spectrum represents the whole “-omics” of a biological sample, FTIR spectroscopy is a promising tool for the development of a clinically useful biomarker which reflects the onset and progression of a disease and ultimately enabling early identification of diseases. Hereby, FTIR spectroscopy has had an important role in the field of disease diagnostics in recent years^16–19^, especially when complemented with Attenuated Total Reflectance (ATR) due to its rapidity and ease to put into clinical practice. Furthermore, different chemometric methods such as Hierarchical Cluster Analysis (HCA) and Principal Component Analysis (PCA) together with FTIR spectral measurements have been successfully used in screening and diagnosis^20^, providing highly sensitive and specific discrimination of diseases based on spectral differences. There is a great deal of literature on the applications of FTIR spectroscopy to neurological diseases such as Alzheimer’s disease (AD)^21–24^, Parkinson’s disease (PD)^25–27^, multiple sclerosis^28–30^, epilepsy^31,32^, bipolar disorder^33^, schizophrenia^33,34^, and depression^35^ in human and animal models. Protein misfolding and aggregation, to which FTIR spectroscopy is sensitive, are the hallmark of a number of neurological diseases^36^. It is now recognized that the aggregation of the amyloid β (Aβ) peptide is responsible for the onset of some of the diseases. FTIR spectroscopy was used to identify the biochemical variations in the plasma of AD patients compared with those of control subjects and oxidative stress-dependent variations in AD plasma were revealed and AD patient serum from non-demented ones were distinguished by hierarchical classification^23^. In another study, it is revealed that plasma samples from cognitive impaired individuals exhibit a higher content of saturated lipids, carboxylic acids, reactive carbonyls, and other molecules related to oxidative stress and protein modifications^21^. A synchrotron FTIR microspectroscopic study aiming to analyze the Lewy bodies (LBs) in the brain of PD patients showed a shift in IR spectrum indicating the abundance of β-sheet-rich structure in LBs^25^. In a recent study, a clear discrimination between the bipolar, schizophrenic and control groups’ blood samples was obtained by FTIR spectroscopy and multivariate analysis methods^33^. All these studies strongly support that FTIR spectroscopy has potential in diagnosing and identifying disease states and may prove to also be of prognostic value^37^.

Demyelination in MS causes significant alterations in FTIR spectrum of the affected areas, reflecting the degradation of the myelin sheath and accumulation of the breakdown products. FTIR has been successfully used in the characterization of the white matter, grey matter and MS plaques from human CNS tissue and identification of the pronounced loss of lipids in MS plaques by using the alterations in the lipid bands^30^. The impact of free radical accumulation in the MS pathogenesis was studied by FTIR spectroscopy on demyelinated MS lesions^29^. The oxidation of protein and lipids at the MS lesions was pointed out, which supports the co-accumulation of free radicals in the pathogenesis of MS lesions. A recent study was designed using experimental autoimmune encephalomyelitis (EAE), animal model for MS, in combination with FTIR microspectroscopy along with artificial neural networks (ANNs) to gain further insight into the cellular and molecular mechanisms involved in the neuropathology of MS^28^. During the course of the disease progression, subtle biochemical and structural alterations throughout the cerebellum and spinal cords of EAE not detected by conventional histological methods were observed by FTIR spectroscopy as an early indication of the clinical signs of EAE^28^. All these studies are limited to tissue sampling.

Although body fluid samples are ideal candidates in diagnosis of neurodegenerative diseases, their analysis using FTIR spectroscopy in this regard is missing. The spectroscopic analysis of body fluids in disease diagnosis is a rather feasible and straightforward method, not only for neurodegenerative diseases but also for cancer, diabetes, and arthiritis^38–42^. Cerebrospinal fluid (CSF) are promising body fluid for the identification and characterization of neurodegenerative diseases. Since its compartment is in close anatomical contact with brain interstitial fluid, CSF may directly reflect the biochemical changes related to neurodegenerative diseases, including MS^43,44^. The fact that many CNS diseases may show numerous discrete lesions similar to those of MS makes its diagnosis difficult. Hence, it is eminent to discover new methods that better discriminate MS from other CNS diseases.

In the present study, ATR-FTIR spectroscopy coupled with multivariate analysis methods were utilized to develop a new procedure for the diagnosis and differentiation of RRMS and CIS patients via cerebrospinal fluid and to determine any differences specifically related to disease progression based on the protein, lipid and nucleic acid profile of diseased groups. Pursuing such an evaluation will be of considerable value, because obtained information will give new insights into the disease progression and thus, point out the potential targets for treatment.

## Results and Discussion

**Figure 1:**
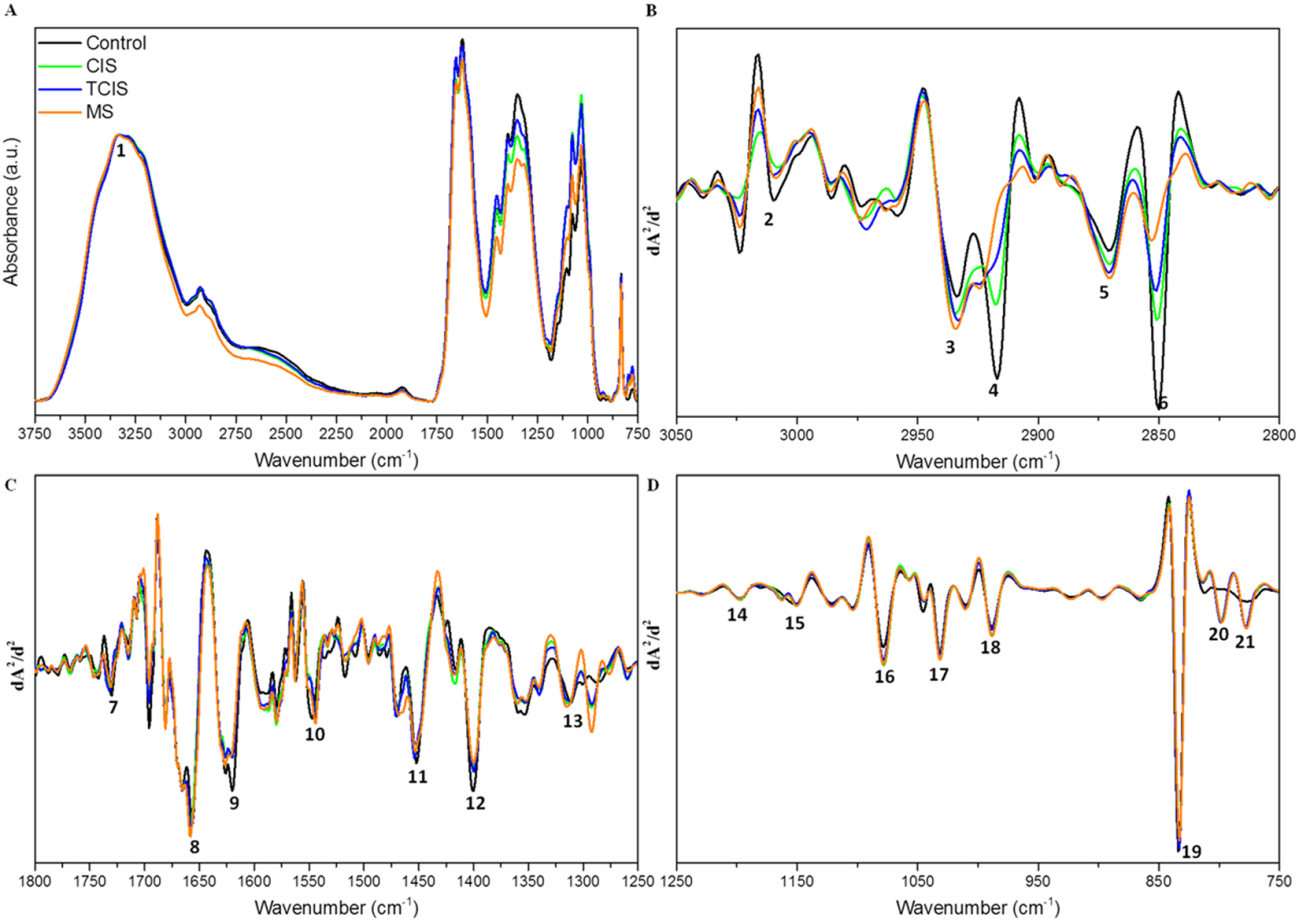
Representative IR spectra of the control, CIS, TCIS and RRMS patients’ CSF samples: (**A**) absorbance spectra in the 3750–750 cm^−1^ region (normalized with respect to the amide A band located at 3330 cm^−1^), (**B**) second derivative vector normalized spectra in the C-H region (3050–2800 cm^−1^), and second derivative vector normalized spectra in the (**C**) 1800–1250 cm^−1^, and (**D**) 1250–750 cm^−1^ ranges of the fingerprint region (the labeled peaks are assigned in Table S-2).

In the current study, we performed ATR-FTIR spectroscopy to characterize and differentiate RRMS and CIS patients by CSF analysis. Figure 1A displays the typical FTIR spectra of the CSF samples for the groups under study in the 3750–750 cm^−1^ spectral region. Second derivative vector normalized FTIR spectra obtained from the control, CIS, TCIS and RRMS samples in different spectral regions, namely 3050–2800 cm^−1^ (C-H region), 1800–1250 and 1250–750 cm^−1^ (1800–750 cm^−1^ fingerprint region) are presented in Fig. 1B,C and D, respectively. FTIR spectra of CSF samples originate in particular from characteristic absorption bands due to the vibrations of various biological molecules. The absorption bands labeled in Fig. 1 and their assignment according to the related literature^37,45,46^ are given in the supplementary material (Table S-2). As seen from Fig. 1, there are obvious spectral differences in the functional groups of the molecules such as lipids, proteins, RNA, DNA, and carbohydrates of the cerebrospinal fluids obtained from RRMS patients compared to the control and CIS patients. Therefore, further investigation of the spectra was focused on the disease-induced changes. Variations in band positions give structural information about the relevant molecule, whereas variations in band intensity or more accurately area under spectral bands are directly proportional to the concentration of the functional groups belonging to the relevant molecule (in accordance with Beer-Lambert law)^47^.

IR spectra consist of two main regions; the C-H and fingerprint regions. C-H region is chiefly associated with lipid spectral bands. The representative second derivative vector normalized spectra of the control, CIS, TCIS and RRMS study groups in the C-H region (3050–2800 cm^−1^) are presented in Fig. 1B. The intense antisymmetric and symmetric CH_2_ stretching vibrations (~2920 and 2850 cm^−1^, respectively) from aliphatic ‒CH_2_ functional groups stem from long hydrocarbon chains in lipids. In addition, there is a unique vibrational frequency of unsaturated lipids at ~3010 cm^−1^ assigned as olefinic C=CH stretching vibration. This band can be utilized for monitoring the content of unsaturated lipids^48–50^.

Remarkable changes in lipid-oriented spectral bands (# 4 and 6) for RRMS can be seen from Fig. 1B. These changes were intensity reduction and wavenumber shifts towards higher wavenumbers in the lipid bands (CH_2_ antisymmetric and symmetric stretching) compared to the control, CIS and TCIS groups (Table 1). Altered CSF lipid content in MS patients, which may provide valuable insights into the diagnosis and pathogenesis of this disorder, has been previously reported in several studies^51,52^. Although some studies have reported an increase in CSF lipids, several others have reported a decrease in CSF cholesterol and lipid transfer proteins in MS patients^53,54^. In our study, there was also a reduction in the intensity of the olefinic =CH stretching band compared to the control as seen from Fig. 1B.

**Table 1:** Changes in the wavenumbers of unsaturated and saturated lipid functional groups, amide I and II bands, and bandwidth values of the amide I vibrations.

Bands	Wavenumber (cm^−1^)			
	Control	CIS	TCIS	MS
Olefinic C=C-H stretching	3008.63 ± 0.40	3008.36 ± 0.49	3009.94 ± 0.84	3008.38 ± 0.39
CH_2_ antisymmetric stretching	2918.09 ± 0.44	2919.01 ± 0.93	2921.47 ± 1.00*	2922.29 ± 0.67****
CH_2_ symmetric stretching	2850.42 ± 0.16	2851.66 ± 0.63	2852.26 ± 0.64*	2852.72 ± 0.43***
Ester C=O stretching	1730.28 ± 0.14	1732.03 ± 0.44**	1732.35 ± 0.35***	1731.20 ± 0.33
Amide I	1657.11 ± 0.25	1657.68 ± 0.33	1657.11 ± 0.33	1658.04 ± 0.22*
Amide II	1546.76 ± 0.39	1544.88 ± 0.49***	1544.04 ± 0.24****	1544.34 ± 0.14 ****
**Bandwidth (cm** ^**−1**^ **)**				
Amide I	8.38 ± 0.29	7.98 ± 0.65	8.08 ± 0.43	10.99 ± 0.63**

The degree of significance for the comparison of the diseased groups with respect to the control group was denoted as *p < 0.05, **p < 0.01, ***p < 0.001, ****p < 0.0001.

**Figure 2:**
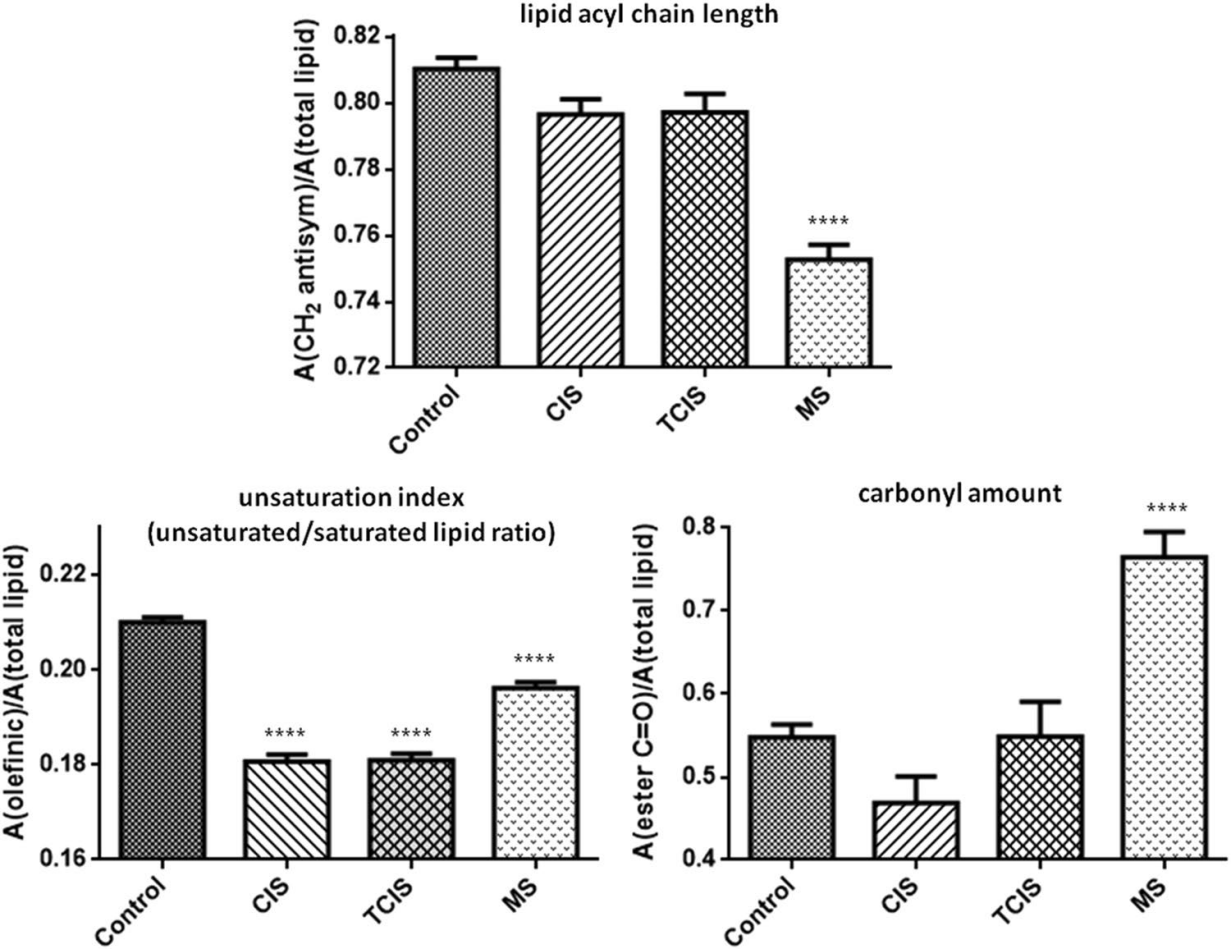
Bar graphs of the CH_2_ antisymmetric/total lipid, olefinic/total lipid, ester carbonyl/total lipid ratios of control and diseased CIS, TCIS, RRMS groups (The degree of significance for the comparison of the diseased groups with respect to the control group was denoted as *p < 0.05, **p < 0.01, ***p < 0.001, ****p < 0.0001).

In order to investigate possible disease-induced variations, the molecular composition and structure of lipids were further analyzed by calculating the area ratios of several specific lipid functional groups to the total lipid content. For this purpose, unsaturated/saturated lipid, carbonyl/lipid and CH_2_ antisymmetric/total lipid ratios were calculated (Fig. 2). Total lipid content was calculated as the sum of CH_2_ antisymmetric and symmetric stretching bands, which correspond to saturated lipids. The carbonyl/lipid ratio, indicating the carbonyl ester concentration in lipids of the system, was calculated by taking the ratio of the area of the carbonyl ester band (~1732 cm^−1^) (peak # 7) to total lipid. The carbonyl/total lipid ratio was significantly higher (p < 0.0001) in the RRMS group compared to other groups. The unsaturated/saturated ratio was calculated by taking the ratio of the area of the olefinic band (~3009 cm^−1^) to total lipid (sum of the saturated lipid bands). The olefinic/total lipid ratio, as an unsaturation index pointing out the content of double bonds in the lipid structure^50,55^, decreased significantly in all the diseased groups compared to the control group (p < 0.0001). The area ratio of CH_2_ antisymmetric stretching band to the total lipid was also calculated to obtain information about qualitative lipid acyl chain length changes. This ratio was quite lower (p < 0.0001) in the RRMS group compared to other groups, revealing the presence of shorter-chained lipids in the RRMS group relative to the control and CIS groups.

Brain contains the second highest lipid content after adipose tissue and almost all brain lipids modify the structure, fluidity and function of cellular membranes. Disease processes result in alterations both in the content and composition of the brain lipids which contain various amounts of saturated, mono**-** and polyunsaturated fatty acids^52,56,57^. The inflammatory environment in demyelinating lesions leads to the generation of oxygen- and nitrogen-free radicals. Thus, oxidative stress is involved in the inflammation leading to demyelination and neurodegeneration in the pathogenesis of MS^58,59^. CNS can be affected from oxidative damage especially by means of polyunsaturated fatty acids which is susceptible to oxidative attack because of their double bond content. Increased degradation of polyunsaturated fatty acids may induce peroxidation of membrane lipids, which is usually accompanied by the formation of degradation products such as aldehydes, shorter-chained lipids and carbonyl compunds^60^. The decrease in the unsaturated/saturated lipid ratio may be due to the decrease in the olefinic content in the diseased groups’ cellular membrane lipids, consistent with increased lipid peroxidation. Moreover, the breakdown of lipid acyl chains was confirmed by the decrease in CH_2_/total lipid ratio of RRMS samples which indicates that the degradation products of lipids contain shorter-chained lipids. These reactions in lipid membranes are usually accompanied by the formation of a wide variety of products, including alkanes and carbonyl compounds. Since free radicals can also oxidize membrane proteins and the oxidation of proteins causes the production of some additional carbonyls^47^, this may also contribute to the increase in the carbonyl ester/total lipid ratio. Hence, the higher carbonyl/lipid ratio that was detected in the RRMS patients also suggests the oxidation of lipids. The oxidation products of lipids have been successfully detected *in vitro* by FTIR spectroscopy and the observed increase in carbonyl groups and degradation of acyl chains is consistent with our study^61^. Lipid peroxidation is of great significance since it modifies the physical properties of membranes, including its permeability to diverse solutes and the packing of lipids and proteins in membranes, which in turn, influences the function of biological membranes^62^.

The lipid acyl chain flexibility (order/disorder state of lipids) information, which is strongly dependent on the composition, hydration, and content of membrane proteins and other factors, can be determined from the variations in the position of the antisymmetric and symmetric CH_2_ stretching bands. For example, a shift to higher wavenumber values implies a higher acyl chain flexibility indicating lipid disordering^63,64^. The wavenumber of the CH_2_ antisymmetric and symmetric stretching bands shifted significantly towards higher values with disease progression in comparison to the control (Table 1). This shift towards higher values implies that lipid order decreases and acyl chain flexibility increases for TCIS and RRMS patients. The bandwidth of CH_2_ antisymmetric or symmetric stretching bands gives information about membrane dynamics, since it is related to the motional rates of the lipid molecule^64,65^. No significant changes in the bandwidth values of CH_2_ antisymmetric stretching bands of diseased groups compared to the control group were observed.

The fingerprint region (Fig. 1C,D) consists of several spectral bands arising from the functional groups of proteins, lipids, carbohydrates and nucleic acids (RNA/DNA). As mentioned previously, the carbonyl amount was significantly higher (p < 0.0001) in the RRMS group compared to the other groups (Fig. 2). The amide I/amide II area and/or intensity ratio and the position of these absorptions are sensitive to protein structural and conformational changes^15^. A highly significant decrease in the wavenumber of the amide II band for all diseased groups and a slight but significant increase in the wavenumber of amide I band for the RRMS group were observed, indicating alterations in protein conformation (Table 1). Furthermore, a significant decrease was observed in the amide I/amide II area ratio for the RRMS group, suggesting alterations in protein structures. Since the band area values of both amide I and amide II bands increased in the diseased groups (data not shown), the decrease in the amide I/amide II area ratio may be due to higher increase in the content of N–H bending and C–N stretching relative to the content of C=O stretching in the proteins of RRMS patients’ CSF samples. Moreover, a significant broadening in the bandwidth of the amide I band (p < 0.01) was observed in the RRMS group in comparison to the control group (Table 1). In a previous study, proteins were thought to be oxidized at active MS lesion sites due to broadening in the amide I band^29^. The formation of additional carbonyls on some amino acid residues results from protein oxidation. It has been proposed that some of these carbonyls reside adjacent to amines and this might lead to a spectroscopic absorption and a broadening in the amide I band^29,47^. Accumulation of carbonylated proteins has been involved in the etiology and/or progression of several neurological disorders including Alzheimer’s disease, Parkinson’s disease, amyotrophic lateral sclerosis, and multiple sclerosis^66^. In a recent study, Sadowska-Bartosz and colleagues aimed to ascertain oxidative stress in RRMS patients without treatment and they observed an elevated level of protein carbonyls in RRMS patients without treatment and confirmed the occurrence of protein oxidative damage in MS^67^.

**Figure 3:**
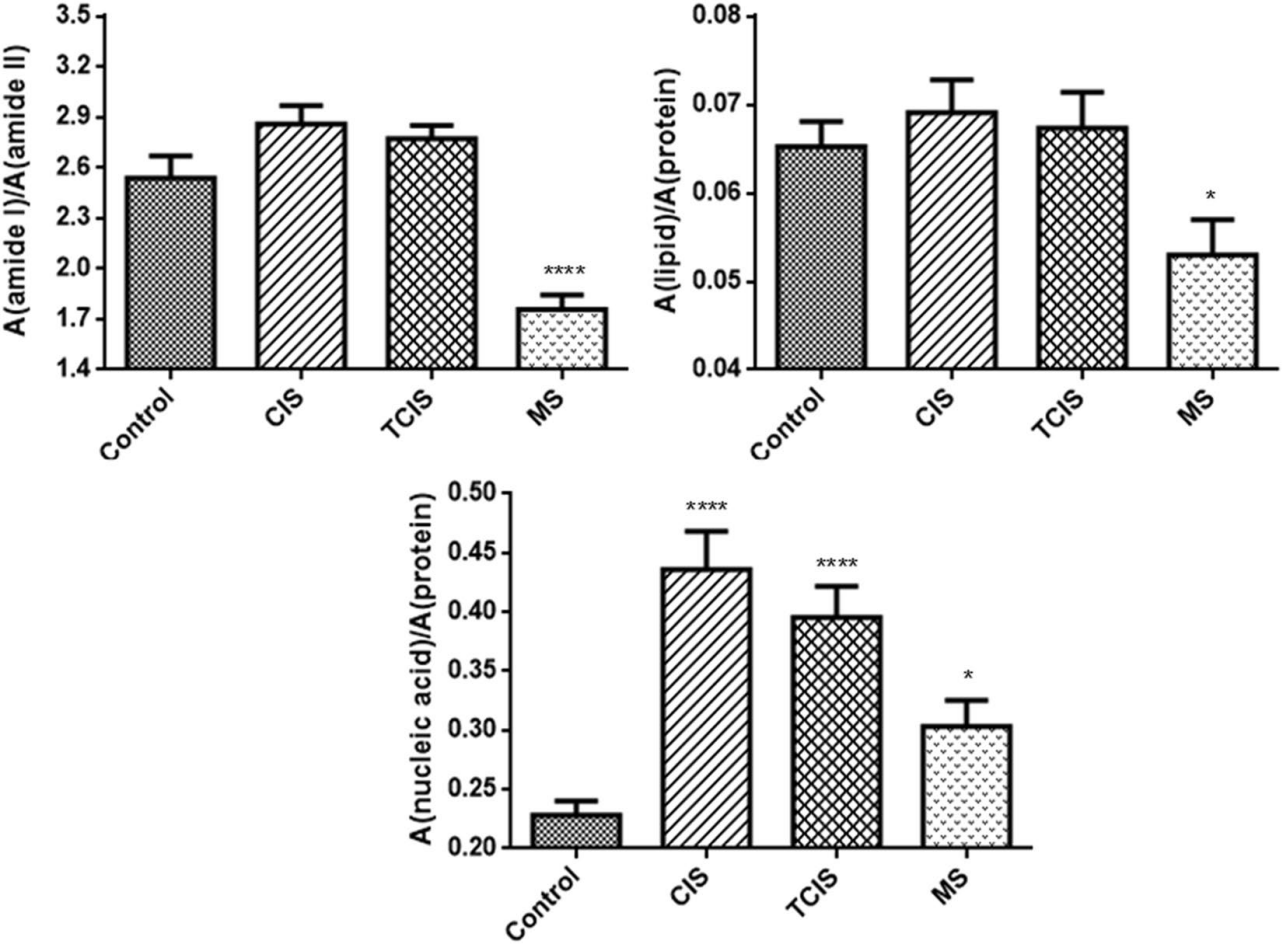
Bar graphs of the amide I/amide II, lipid/protein and nucleic acid/protein area ratios of control and diseased CIS, TCIS, RRMS groups (The degree of significance for the comparison of the diseased groups with respect to the control group was denoted as *p < 0.05, **p < 0.01, ***p < 0.001, ****p < 0.0001).

The lipid to protein ratio can be obtained by taking the ratio of the areas of the bands arising from lipids and proteins. The ratio of the sum of the area under the CH_2_ antisymmetric and symmetric stretching bands to the sum of the area under the amide I and II bands was used to evaluate the lipid to protein ratio (Fig. 3). As seen from Fig. 3, the lipid/protein ratio decreased significantly in the CSF samples of the RRMS patients (p < 0.05) compared to the control group. This decrease might be attributed to a lower lipid and/or higher protein content, indicating an alteration in the lipid and protein metabolism in the RRMS group.

FTIR spectra of nucleic acids show a number of characteristic bands. As in other biomolecules discussed above, the integrated area of these bands also provides information about the concentration of nucleic acids. Nucleic acid/protein ratios of the control and diseased CIS, TCIS, RRMS groups are shown in Fig. 3 and the observed increase in this ratio of the diseased groups suggests an increased DNA/RNA content in the CSF samples of the patients. The ratio of the integrated absorbance values of the bands at 1080 and 1540 cm^−1^ were proposed as a clinical parameter for the evaluation of the degree of malignancy in patients affected by B-chronic lymphatic leukaemia^68^. In a study with experimental autoimmune encephalomyelitis (EAE), an animal model for MS, IR images of cerebellum tissues collected during the progression of the disease confirms the dramatic decrease in lipids located within sites of the MS lesions and the formation of MS lesions accompanied by an increased content of nucleic acids also observed in our human study. Furthermore, similar to our results, in that animal model study they proposed lipid, protein, and nucleic acid bands as spectral markers for MS with the use of PCA and ANNs^28^.

**Figure 4:**
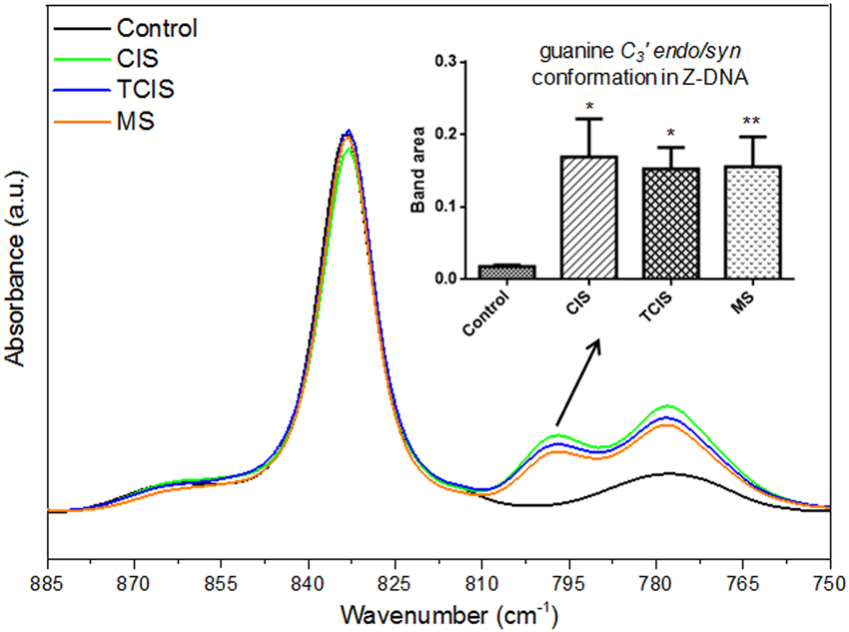
IR absorbance spectra of CSF samples from control, CIS, TCIS and RRMS patients in the 885–750 cm^−1^ spectral range normalized with respect to the amide I band located at 1657 cm^−1^ and changes in the band area of the band related to Z conformation DNA (795 cm^−1^) in subpanel (The degree of significance for the comparison of the diseased groups with respect to the control group was denoted as *p < 0.05, **p < 0.01).

Figure 4 shows the 885-750 cm^−1^ spectral region which includes two DNA related vibrations at 832 and 795 cm^−1^ that can be attributed to the B-form helix conformation of DNA and guanine in a *C*_3_*′ endo/syn* conformation, which is present in the Z conformation of DNA, respectively^45,69^. There was no significant change in the intensity or area of the B-form helix conformation of DNA, while there were quite significant changes for the Z conformation of DNA. The Z conformation of DNA band was not observed in the control CSF samples contrary to the diseased samples (Fig. 4). The integrated area of this band indicated increased content of Z conformation of DNA in the diseased groups which indicates conformational changes in native DNA with a tendency towards the formation of local left-handed Z structure. It is inferred that the changes in the Z conformation of DNA and its absence in the control CSF samples remarkably contribute to the discrimination of the diseased group from the controls and have potential diagnostic value.

**Figure 5:**
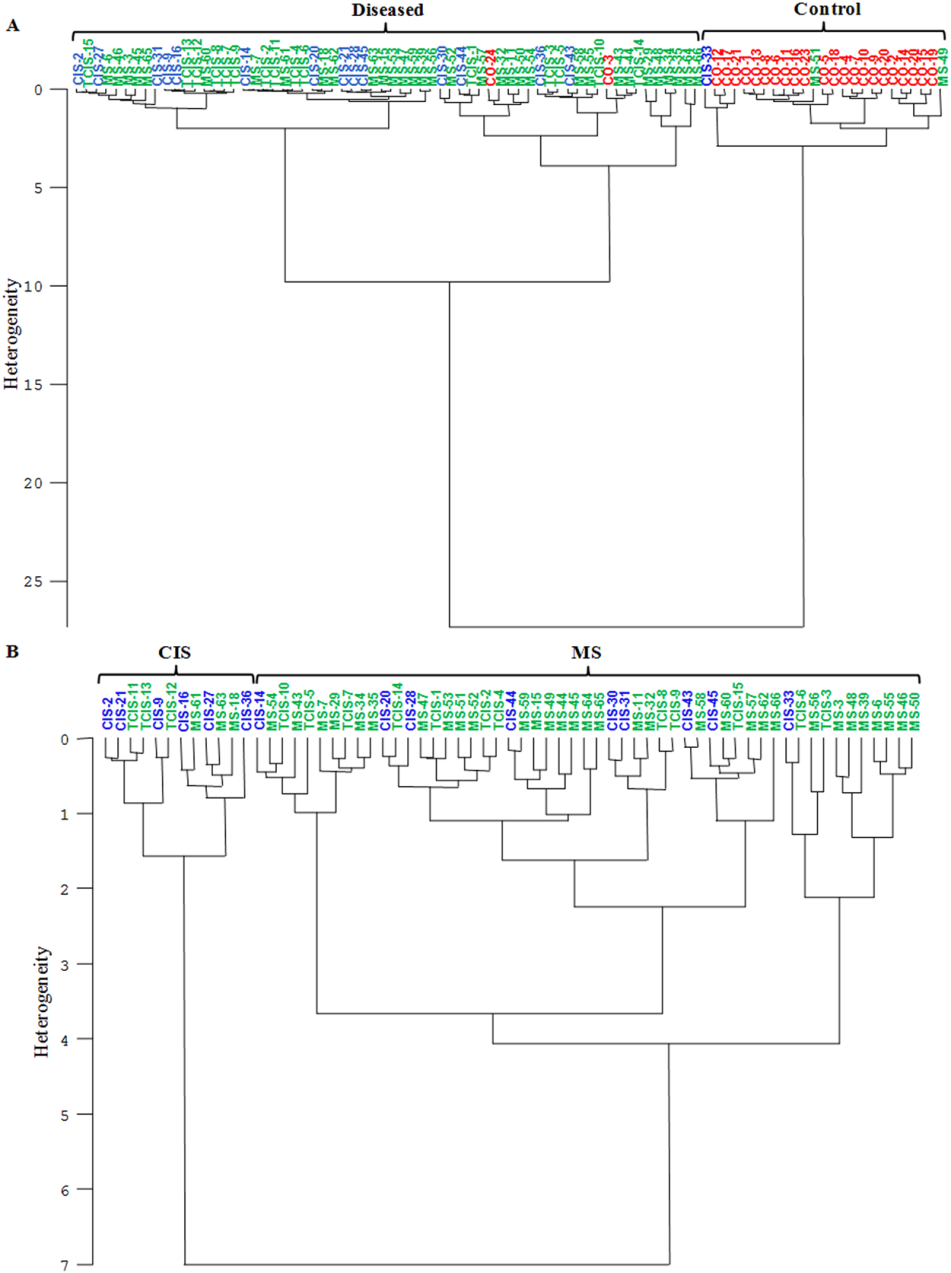
Hierarchical clustering (HCA) of CSF samples for (**A**) CO, CIS, TCIS and RRMS groups in the 815–785 cm^−1^ spectral region, and (**B**) CIS, TCIS and RRMS groups in the 3025–2800 cm^−1^ spectral region from second derivative vector normalized IR spectra.

In addition to the spectral characterization studies mentioned above, meaningful diagnostic information can be obtained rapidly with the application of multivariate analysis methods to infrared spectra. In this respect, unsupervised multivariate analysis methods such as HCA and PCA were performed to different spectral regions to determine the most suitable spectral ranges that can be used as a marker for relapsing-remitting multiple sclerosis diagnosis. HCA was firstly applied to second derivative vector normalized IR spectra to differentiate diseased groups from the control and from each other. The best results were achieved in the C-H and 815–785 cm^−1^ spectral regions. Figure 5A and B shows the HCA results of the control vs. diseased groups in the 815–785 cm^−1^ spectral region and CIS vs. RRMS (including TCIS) groups in the C-H spectral region (3025–2800 cm^−1^), respectively. The dendrograms indicate a notable clustering of the groups under study. As seen from Fig. 5A, successful differentiation was obtained between the control and two different disease groups in the 815–785 cm^−1^ spectral region. The magnitude of similarity is the heterogeneity values in cluster analysis. Higher heterogeneity between the clusters demonstrates higher dissimilarity among analyzed groups. The highest heterogeneity greater than 25 was observed in the differentiation of the control and diseased groups (Fig. 5A). When TCIS and RRMS groups were considered together, the HCA results revealed that most of the RRMS and TCIS samples were successfully clustered in the same group. The heterogeneity value for the differentiation of CIS and RRMS group was observed as 7. In order to measure the efficiency of the discrimination, sensitivity and specificity values based on the obtained clusters were calculated. The sensitivity and specificity values (95 and 92%, respectively) indicate much better discrimination of the diseased groups from the controls when compared to those (88 and 40%, respectively) of the diseased groups from each other. As can be seen from Fig. 5B some CIS samples were also clustered in RRMS group. These samples should be followed up because they may transform to RRMS over time. This also causes to the low specificity value for the differentiation of RRMS and TCIS groups from the CIS group.

**Figure 6:**
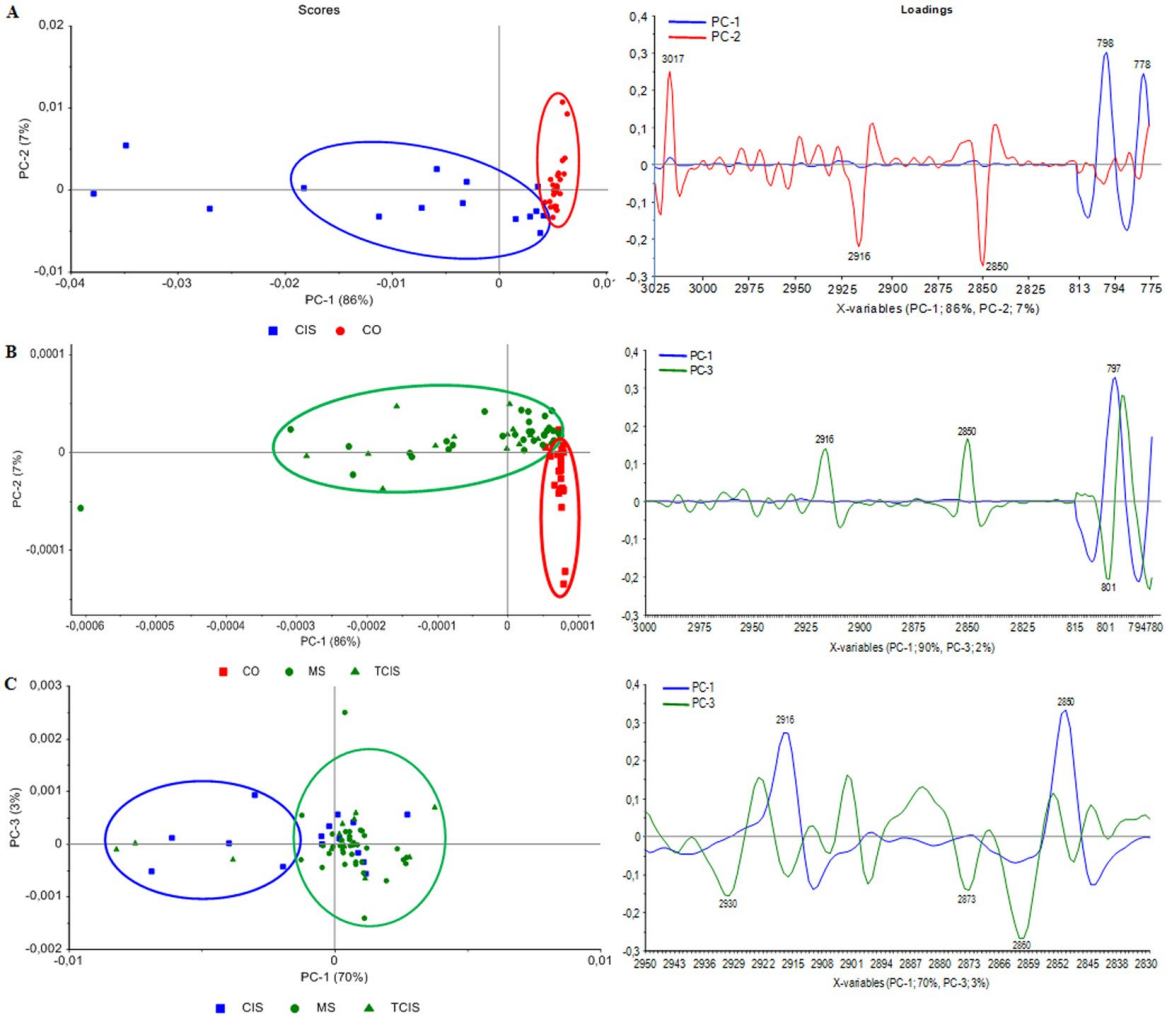
PCA scores and loading plots for FTIR spectra of (**A**) Control and CIS samples in the 3025–2800 and 813–775 cm^−1^ spectral regions, (**B**) Control, RRMS and TCIS samples in the 3000–2800 and 815–780 cm^−1^ spectral regions and (**C**) CIS, RRMS and TCIS samples in the 2950–2830 cm^−1^ spectral region.

The clustering of the spectra obtained from the control and diseased groups was further analyzed by PCA. PCA results are presented as score and loading plots. Scores plot displays the distribution of samples in the PC space and the loading plots identify the regions of the spectrum which are responsible for the clusters appearing in the scores plot of CSF samples. The positive and negative peaks observed in the loading plots indicate that these peaks strongly affect the principal components and so contribute to the discrimination of the groups under study. PC analysis was applied to mean-centered, second derivative and vector normalized IR data. Firstly, mean-centered PCA was conducted over the range of 4000–650 cm^−1^ for all groups and their loading plots are given in supplementary material (Fig. S-1). These plots clearly showed that the spectral differences between the groups occurred dramatically in the 3025–28000 cm^−1^ and 850–750 cm^−1^ regions. PCA score plots and the corresponding loading plots of these spectral ranges are given in Fig. 6. For the separation of the diseased groups from the control group, the best clustering was found by using the combination of the C-H region and the region related to Z conformation of DNA. The PCA score plots shown in Fig. 6A and B demonstrated that the clusters of CIS and RRMS (TCIS included) samples were clearly separated from the control group. The evaluation of the scores plot for control-CIS and control-RRMS (TCIS included) groups demonstrated 86% of the variation to be accounted by the first principal component (PC-1) (Fig. 6A,B). The clusters of CIS were distinguished from the control along PC-2 (Fig. 6A). The clusters of RRMS (TCIS included) were distinguished from the control along PC-1 and PC-3 (Fig. 6B). For the separation of the diseased groups from each other, a part of the C-H region (2950–2830 cm^−1^) was used. The PCA score plot shown in Fig. 6C indicated that the clusters of CIS and RRMS (TCIS included) groups differentiated from each other with a slight overlapping of CIS samples in the RRMS group. Moreover, the RRMS cluster (TCIS included) was distinguished from the CIS group along PC-1 (Fig. 6C). The evaluation of the scores plot for CIS-RRMS (TCIS included) groups demonstrated 70% of the variation to be accounted by the first principal component (PC-1) (Fig. 6C). The obtained notable biomolecular changes in the lipids and Z conformation of DNA from spectral evaluation enables the discrimination of the groups from each other.

The leave-one-out type cross validation (LOOCV) was performed for all PCAs in the present study. The obtained score plots from the calibration and cross validation sets were compared so that prediction errors are kept at the minimum level^70^. The PCA score plots obtained from the calibration set (blue) and the corresponding leave-one out cross validation (red) for the groups under study are given in supplementary material (Fig. S-2). Since the calibration and cross validation results were very close to each other, it can be said that the PCA model obtained is reliable.

**Figure 7:**
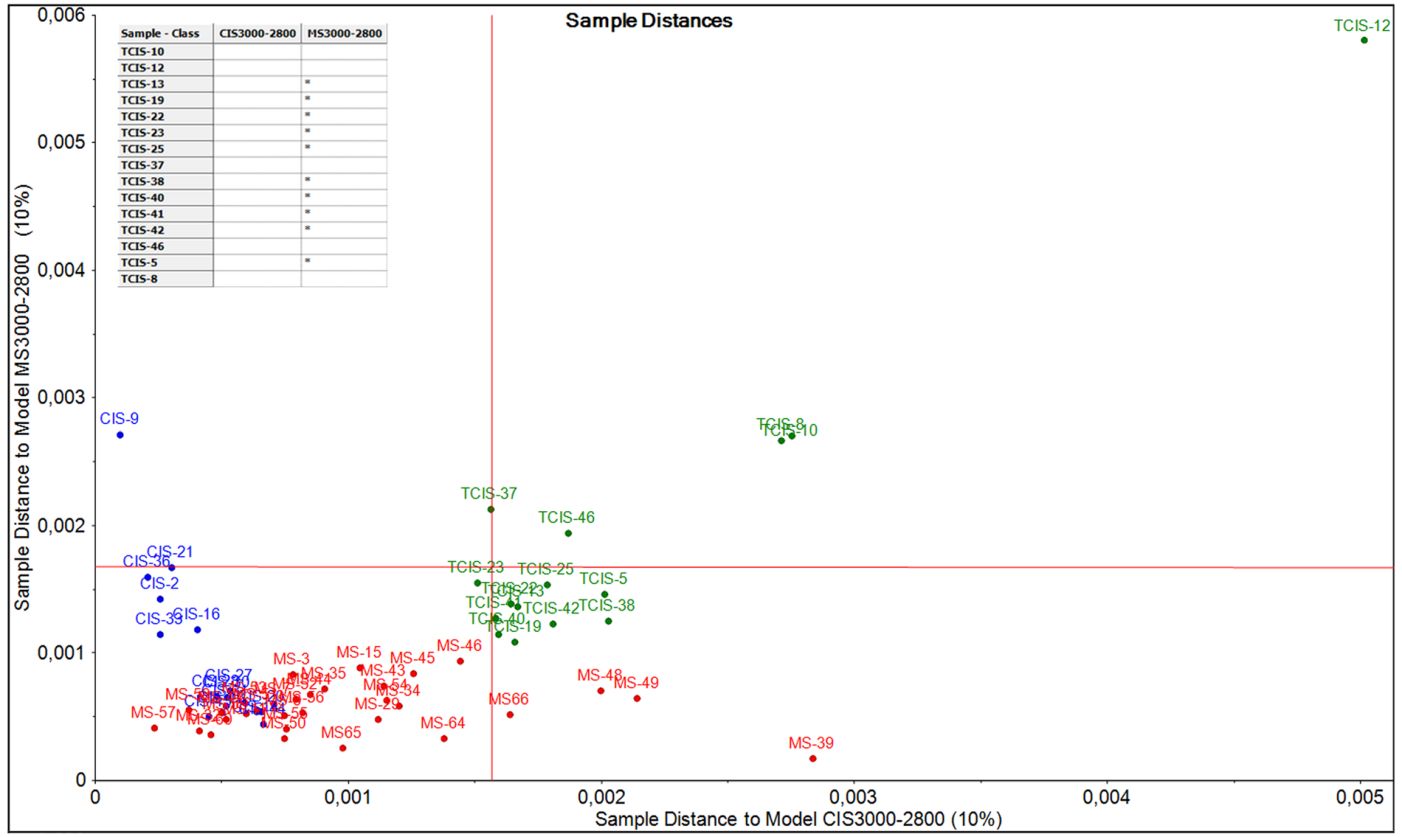
Cooman’s plot of RRMS (red), CIS (blue) and TCIS (green) samples and the classification table of TCIS samples at 10% significance level.

In order to identify local models for different classes and to predict a probable class membership for new observations, Soft Independent Modeling of Class Analogy (SIMCA) approach, a supervised classification tool, was performed. For this classification process, the second derivative vector normalized pre-processed data was used in the C-H spectral region (3000–2800 cm^−1^) following the discrimination results obtained by PCA. Prior to SIMCA classification, PCA models for CIS (n = 15) and RRMS (n = 35) classes were developed as the training data set. CIS samples in the training data set were chosen from CIS samples which are diagnosed as CIS by neurological examinations. Subsequent to the set up of models, SIMCA classification was carried out for the test group (TCIS samples) and the residual distances were calculated. A test sample is then assigned to a certain class, if the residual distance to the particular model is below the statistical limit for that class. If the residual distance of a sample exceeds the upper limit for every class in the data set, the sample would not be assigned to any of the classes, since it is considered either an outlier or a result of a class not represented in the data set^71,72^. The Cooman’s plot, which enabled the sample-to-model distances to be plotted against each other for the two models (RRMS and CIS models) at 10% significance level, and the classification table of TCIS group at the same significance level are shown in Fig. 7. Cooman’s plot can be seen in this figure and a model distance greater than 8 indicates that the two models are quite different and the groups are distinguished. However, a relatively slight overlapping occurred, indicating some common characteristic parameters between CIS and RRMS groups. This result may indicate that some of the CIS patients may transform to MS over time. It seems from Cooman’s plot and the classification table that 10 of 15 patients from the TCIS group were classified as RRMS at a 10% significance level and the other 5 patients fell outside the statistical limits (in the upper right corner) which means that these 5 patients belong to neither of the models^72^. This indicates that these samples belong to a class that has not been used in the classification or they may simply be outliers. The model was created by assuming that all the samples in the CIS model were exactly CIS. Since it is known from the results of this study that a total of nine CIS patients will develop RRMS over time, SIMCA classification may be clearly affected by these outliers and even incorrectly classify a number of good observations.

**Figure 8:**
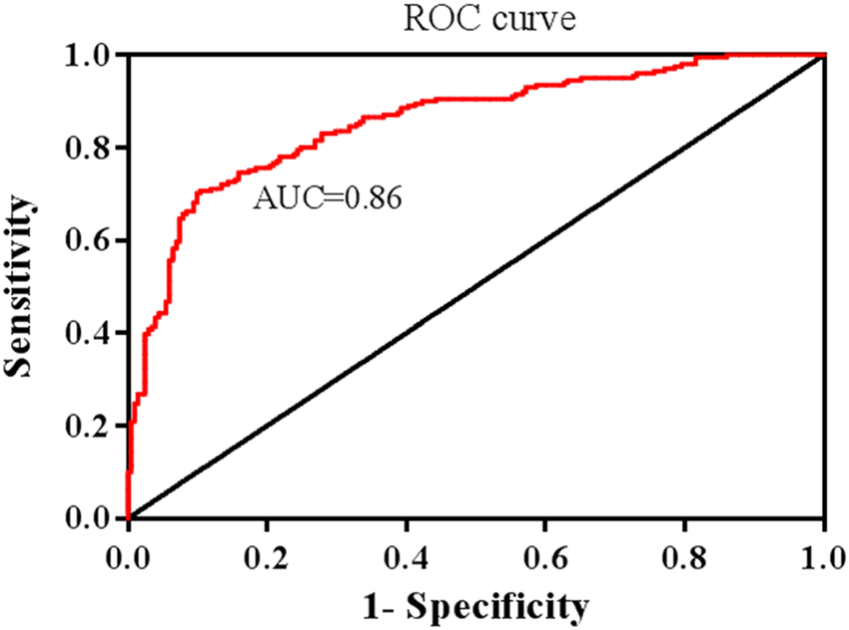
ROC curve obtained from the classification test results based on infrared spectra.

The classification model performance can be visualized by the plot of Receiver Operating Characteristic (ROC) curve that shows how the true positive rate changes with the false positive rate. Since the classification methods produce probability values representing the degree to which class the objects belong, the statistics like true and false positive rates can be directly obtained from the outputs of the method. The resulting ROC curve is given in Fig. 8. The obtained area under the ROC curve (AUC) value was 0.86 (95% confidence interval [CI], 0.82–0.89) (p < 0.0001), indicating that CIS and RRMS patients are well separated with this classification model. The AUC value is quite similar to those of MRI results from MS patients given in the literature by other groups, where the reported values are 0.82 and 0.83^73,74^. The results of the present study reveal that, in MS diagnosis and CIS to RRMS transformation, due to its low cost, rapidity and sensitivity, infrared spectroscopy can be used as a complementary technique to MRI which is a golden standard.

## Conclusion

The results of the current study demonstrated that the biomolecular composition and structure of CSF samples for diseased groups (MS and CIS) differs from the control samples. The biomolecular differences such as disease-induced peroxidation of membrane lipids, protein oxidative damage and increased content of nucleic acids have diagnostic significance and enable the discrimination of diseased CSF samples from each other. For example, significant increase in carbonyl amount and significant decrease in amideI/amide II and lipid/protein ratios were observed only for RRMS groups and, therefore can be used as diagnostic biomarkers for accurate diagnosis of RRMS groups. The observation of a new band in the diseased groups located at 795 cm^−1^ belonging to guanine C3′-endo/syn conformation in the Z-DNA that is non-existent in the control CSF samples was an important spectral output of the present study. Therefore, it has been shown that this band can be used as a biological marker for the diagnosis of RRMS and CIS from individuals that have no neurological disorders. A very successful separation of TCIS and RRMS groups from the CIS group with high sensitivity was obtained by HCA and PCA scores. Moreover, 10 of 15 TCIS samples were accurately identified by SIMCA analysis. A total of nine CIS samples were consistently clustered in the multiple sclerosis class which may be an indication that these CIS patients may also transform to MS over time as we have observed in a follow-up process. This suggests that FTIR spectroscopy coupled with multivariate analysis can be used in early diagnosis of RRMS.

It is advised that patients should be treated as early as possible after a first clinical demyelinating event (FCDE) and previous studies have shown that early treatment with first stage therapeutics has beneficial effects in patients with a FCDE for reducing the risk of developing MS. Early diagnosis of CIS patients who may definitively convert to MS is important, because time of conversion to clinically definite MS (CDMS) will be postponed by early treatment of the correct patient.

In conclusion, computational and statistical analyses of FTIR spectra might reflect the disease-induced changes long before they become visible to the neurologist.

## Material and Methods

### Patients and sample collection

All participants provided written informed consent and all experimental protocols were approved by the Izmir Tepecik Education and Research Hospital Local Ethics Committee (Approval No. 2013-24). All methods were performed in accordance with the relevant guidelines and regulations of the ethical committee approvals. A total of 24 cerebrospinal fluid (CSF) samples from control individuals and 65 CSF samples from relapsing-remitting multiple sclerosis (RRMS, n = 35) and clinically isolated syndrome (CIS, n = 30) patients, who fulfilled the criteria defined by McDonald^75^, were collected. Of the 30 patients who were diagnosed with CIS, it was determined that 8 (26,66%), 6 (20%), 6 (20%), 6 (20%) and 4 (13,33%) had hemispheric, brain stem, spinal, optic and cerebellar symptoms, respectively. CIS patients were carefully diagnosed and selected via magnetic resonance imaging (MRI) examination, by making sure that there were no lesions in the central nervous system of those patients who had optic neuritis and there was a single lesion clarifying the occurrence of hemispheric, brain stem, cerebellar and spinal symptoms. The number and localisation of T2 hyperintense lesions on T2-weighted FLAIR MRIs in patients with RRMS were fulfilling the 2010 revised McDonald’s criteria. Control individuals were patients treated for orthopedic problems such as meniscus tears, femur fractures, however they were not diagnosed with any neurological disease. Expanded disability status scale (EDSS) and demographic data of the groups under study are given in Table 2.

**Table 2:** Demographic and EDSS data of the groups under study.

Patient and Control Samples	Age	Gender (M/F)	EDSS Mean ± SD
RRMS (n = 35)	34.08 ± 8.20	6/29	2,22 ± 0.83
CIS (n = 30)	32.00 ± 10.73	11/19	1,08 ± 0,19
TCIS: CIS → RRMS (n = 15)	33.71 ± 11.05	6/9	1,78 ± 0,82
Control (n = 24)	36.80 ± 13.35	14/10	—

Oligoclonal bands (OCBs) were investigated in the CSF of all control and diseased patients by isoelectric focusing technique. OCBs positivity (type 2 and 3) was detected in 46% and 77% of the CIS and MS groups’ patients, respectively and in none of the control group patients. The patients were followed up for 24 months. After the follow-up period, 15 CIS patients developed RRMS which was denoted as transformed CIS (TCIS). OCBs were negative in 4 of these patients.

In order to collect CSF, the lumbar puncture technique was used. A needle was inserted into the space between two vertebrae in the lower (lumbar) spine and then it was carefully moved into the CSF filled space surrounding the spinal cord. Approximately 3 milliliters CSF was dripped out into a vial when the needle was in place. The collected CSF samples were then centrifuged for 10 min at 1500 rpm and the supernatant was removed. The centrifuged CSF samples were stored at −80 °C until FTIR experiments and their spectra were acquired within 2 months.

### ATR-FTIR spectroscopy

Frozen CSF samples were thawed in an ice-filled box before the measurements and afterwards they were directly put on the ATR crystal and scanned. ATR-FTIR spectra of all samples were collected by one-bounce ATR mode of a Perkin Elmer Spectrum100 spectrometer (Perkin Elmer Inc., Norwalk, CT, USA) equipped with a Universal ATR accessory. 2 µl of the CSF samples were placed on a Diamond/ZnSe crystal plate and dried with a mild nitrogen gas flux for 5 min to remove excess unbound water. This process was repeated two more times (a total of 6 µl of CSF) in order to get reasonable absorbance values. The IR spectra of the samples were scanned in the 4000–650 cm^−1^ wavenumber region and 200 scans were taken for each interferogram with 4 cm^−1^ resolution at room temperature. The spectrum of air prior to sample spectra acquisition was recorded as a background under identical conditions as the samples and subtracted automatically from all the spectra to eliminate the effects of water molecules in the air. Recording the spectra and data manipulations were performed with Perkin Elmer Spectrum software version 10.03.06. From each sample three randomly taken independent samples (6 µl of CSF each) were prepared and then scanned to check the reproducibility of the identical spectra. The average spectra of these replicates were used in further data analysis. These average spectra were baseline corrected and then normalized with respect to the amide A band for visual demonstration. The band positions were measured according to the center of mass. The bandwidth values of the CH_2_ asymmetric stretching band were measured at 75% of height of the peaks’ maximum from baseline corrected spectra.

### Multivariate Analysis Methods

Unsupervised multivariate analysis methods, namely PCA and HCA, were applied to discriminate the samples based on spectral differences and SIMCA, a supervised multivariate analysis method, was performed to classify the clinically isolated syndrome samples which transformed to relapsing-remitting multiple sclerosis.

The obtained FTIR data for the CSF samples under study were imported into The Unscrambler X 10.3 (CAMO Software Inc., Oslo, Norway) and PCA was applied to mean-centered, second derivative and vector normalized data. Initially, mean**-**centered PCA was conducted over the range of 4000–650 cm^−1^ for all groups and the best spectral regions were decided from the loading plots in the 4000–650 cm^−1^ region as 3025–2800 and 815–775 cm^−1^ for the discrimination of the groups. PCA results are presented as score and loading plots.

For the determination of spectral differentiation between groups under study, HCA was performed by OPUS 5.5 software (Bruker Optics GmbH). The analyses were performed on second derivative vector normalized spectra that were smoothed nine points with the Savitzky–Golay algorithm^76^. The results are displayed as a dendrogram constructed using Ward’s algorithm for hierarchical clustering in two dimensions. In order to evaluate the success of discrimination, the sensitivity and specificity values were calculated from hierarchical clustering of FTIR spectra in different spectral ranges by using the equations given in the supplementary material (Table S-1). While the sensitivity measures the proportion of actual positives which are correctly identified, the specificity measures the proportion of negatives which are correctly identified^77^.

In order to identify local models for possible groups and to predict a probable class membership for new observations, SIMCA approach, a supervised classification technique, was performed using The Unscrambler X (CAMO Software, Inc.) program. SIMCA results are given by sample-to-model distances plot and classification table of test groups. Detailed information about multivariate analysis methods is given in the supplementary material.

### Statistics

The results were expressed as mean ± standard error of mean (SEM). The data were evaluated using a normality test to decide whether the parametric or nonparametric statistical test should be used. Since the data showed normal distribution, RRMS and CIS groups versus the control group were analyzed using the one-way ANOVA and Dunnett’s multiple comparison tests in GraphPad Prism 6 (GraphPad Software, Inc.). p < 0.05 was considered as statistically significant. The degree of significance for the comparison of the diseased groups with respect to the control group was denoted as *p < 0.05, **p < 0.01, ***p < 0.001, ****p < 0.0001.

ROC curve analysis, performed with GraphPad Prism 6 (GraphPad Software, Inc.), was used to assess the predictive ability of the classification methods employed. ROC curve, the true positive rate (i.e., sensitivity) versus false positive rate (i.e., 1-specificity), was plotted using the statistical data obtained by classification method based on IR spectroscopic data. The area under the ROC curve (AUC) is a measure of classification model performance that indicates a successful classification model, if it is close to 1.

## Electronic supplementary material

Supplementary Material
