# Supplementary material for: Relapsing-Remitting Multiple Sclerosis diagnosis from cerebrospinal fluids via Fourier transform infrared spectroscopy coupled with multivariate analysis
Source: Sci Rep. 2018 Jan 18;8:1025. doi: 10.1038/s41598-018-19303-3 (PMC5773569; doi:10.1038/s41598-018-19303-3)
Supplement: Supplementary file 1 — Supplementary Material [file 41598_2018_19303_MOESM1_ESM.doc]

**Relapsing-Remitting Multiple Sclerosis diagnosis from cerebrospinal fluids via Fourier transform infrared spectroscopy coupled with multivariate analysis**

Dilek Yonar^1^, Levent Ocek^2^, Bedile Irem Tiftikcioglu^2^, Yasar Zorlu^2^, Feride Severcan^1,3*^

^1^Middle East Technical University, Department of Biological Sciences, 06800, Ankara, Turkey

^2^Izmir Tepecik Education and Research Hospital, Neurology Clinic, 35180, Izmir, Turkey

^3^Altinbas University, Faculty of Medicine, Biophysics Department, 34147, Bakirkoy, Istanbul, Turkey

***Corresponding Author:**

Feride SEVERCAN

Altinbas University

Faculty of Medicine

Biophysics Department

34147, Bakirkoy, Istanbul, Turkey

Phone: +90 212 709 45 28/5262

Fax: +90 212 445 81 71

e-mail: [feride@metu.edu.tr](mailto:feride@metu.edu.tr)

**Multivariate Analysis Methods.**

*Unsupervised multivariate analysis methods.* Principle Component Analysis (PCA) is a potent data reduction technique that transforms the coordinate system of multi dimensional data sets produced by analytical measurements (FTIR spectra) into a coordinate system representing the orthogonal directions of the largest variances within the data set.^1^ This corresponds to the reduction of a large scale of spectra consisting of thousands of absorbance values to a single point in a multidimensional space using a linear transformation. The coordinates are the principle components (PC), the values that the spectra have in the PC coordinate system are called scores and the plot obtained is called the scores plot. PCs identify the spectral variability among samples in decreasing order.^2,3^ Since PCA is a transformation of the old coordinate system (peaks) into the new coordinate system (PCs), the correlation between the component and original variables is called the loadings. The higher the loading of a particular peak onto a PC, the more it contributes to that PC.^3^

For the determination of spectral differentiation between groups under study, hierarchical cluster analysis (HCA) was performed. In this method, the similarities between the spectra are determined by using distance calculation and classification algorithms. The results are displayed as dendrograms constructed using Ward’s algorithm for hierarchical clustering in two dimensions by graphical means.^4^ Pearson’s correlation coefficients were used to calculate the spectral distances between pairs of spectra. Euclidean distance was used to calculate sample similarities. The magnitude of similarity is the heterogeneity values. Higher heterogeneity between clusters demonstrates higher dissimilarity among analyzed groups.^5–7^

**Table S-1.** Sensitivity and specificity definitions for hierarchical cluster analysis based on FTIR data.

|  | **Positive*** | **Negative*** |  |
| --- | --- | --- | --- |
| Diseased | A | B | Sensitivity = A / (A+B) |
| Control | C | D | Specificity = D / (C+D) |

* Positive and negative values are deduced as follows;

A: number of diseased samples clustered in diseased group

B: number of diseased samples clustered in control group

C: number of control samples clustered in diseased group

D: number of control samples clustered in control group

*Supervised multivariate analysis method.* Soft Independent Modeling of Class Analogy (SIMCA) approach, a supervised classification technique, was performed to identify local models for possible groups and to predict a probable class membership for new observations. Since SIMCA is based on PCA modeling, at first, [PCA](http://www.camo.com/resources/principal-component-analysis.html) is performed on each class in the whole dataset to estimate local models and subsequently, unclassified samples are classified to one of the local models on the basis of their best fit to the relevant model.^8–10^ SIMCA analysis provides additional information (i.e. model distance and Cooman's plot) to the usual PCA results. Model distance plot shows how different two models are from each other with respect to the PC space. A model distance greater than or equal to 3 shows that the two models are quite different, which implies well distinguished classes from each other. Cooman’s plot enables the sample-to-model distances to be plotted against each other for two models. As it includes class membership limits for both models, it is possible to see whether a sample belongs to one class, or both, or none. If the distance calculated for a specific class at a suitable significance levels is lower than the critical distance, then the sample belongs to that class.^3,11^

**IR Band Assignment**

**Table S-2.** General IR band assignments of cerebrospinal fluid.

| **Band No** | **Wavenumber**  **(cm^-1^)** | **Assignment** |
| --- | --- | --- |
| **1** | 3330 | Amide A: protein (N–H stretching) |
| **2** | 3009 | Olefinic =CH stretching vibration: unsaturated lipids, cholesterol esters |
| **3**  **4** | 2934  2917 | CH_2_ antisymmetric stretching (mainly from methylene groups of lipids) |
| **5** | 2873 | CH_3_ symmetric stretching: mainly protein |
| **6** | 2850 | CH_2_ symmetric stretching (mainly from methylene groups of lipids) |
| **7** | 1732 | Ester C=O stretching (triglyceride, cholesterol esters) |
| **8** | 1654 | Amide I: protein (80% C=O stretching, 10% N–H bending, 10% C–N stretching) |
| **9** | 1625 | Mainly protein and nucleic acids contribution due to the base carbonyl stretching and ring breathing mode, β sheet structures |
| **10** | 1545 | Amide II: protein (60% N–H bending, 40% C–N stretching) |
| **11** | 1452 | CH_3_ antisymmetric bending (lipids and proteins) |
| **12** | 1397 | COO^-^ symmetric stretching: fatty acids and aminoacids |
| **13** | 1311 | Amide III: C–N stretching and N–H bending |
| **14** | 1152 | Glycogen absorption due to C–O and C–C stretching and C–O–H deformation motions |
| **15** | 1105 | P–O–C symmetric stretching: RNA |
| **16** | 1075 | PO_2_^-^ symmetric stretching (mainly from nucleic acids) |
| **17** | 1031 | C-O stretching in polysaccharides (strong contribution from glycogen) |
| **18** | 988 | C-O stretching (mainly from RNA) |
| **19** | 832 | B-form helix conformation of DNA |
| **20** | 795 | Guanine in a *C_3_' endo/syn* conformation in the Z conformation of DNA |
| **21** | 776 | Out-of-plane bending vibrations |

**Figure S-1.** PCA scores and loading plots for FTIR spectra of control, CIS, RRMS and TCIS samples in whole spectral region.

**Leave-one-out cross validation results for FTIR spectra**

**Figure S-2.** Leave-one-out cross validation results for FTIR spectra of A) Control and CIS samples in the 3025-2800 and 813-775 cm^–1^ spectral regions, B) Control, RRMS and TCIS samples in the 3000-2800 and 815-780 cm^–1^ spectral regions and C) CIS, RRMS and TCIS samples in the 2950-2830 cm^–1^ spectral region.

**REFERENCES**

1. Wang, L. & Mizaikoff, B. Application of multivariate data-analysis techniques to biomedical diagnostics based on mid-infrared spectroscopy. *Anal. Bioanal. Chem.* **391,** 1641–1654 (2008).

2. Nieuwoudt, H. H., Prior, B. A., Pretorius, I. S., Manley, M. & Bauer, F. F. Principal Component Analysis Applied to Fourier Transform Infrared Spectroscopy for the Design of Calibration Sets for Glycerol Prediction Models in Wine and for the Detection and Classification of Outlier Samples. *J. Agric. Food Chem.* **52,** 3726–3735 (2004).

3. Esbensen, K. H. *Multivariate Data Analysis: In Practice: an Introduction to Multivariate Data Analysis and Experimental Design*. (CAMO Software, 2010).

4. Ward, J. H. Hierarchical Grouping to Optimize an Objective Function. *J. Am. Stat. Assoc.* **58,** 236–244 (1963).

5. Demir, P., Onde, S. & Severcan, F. Phylogeny of cultivated and wild wheat species using ATR–FTIR spectroscopy. *Spectrochim. Acta Part A Mol. Biomol. Spectrosc.* **135,** 757–763 (2015).

6. Gautam, R., Vanga, S., Ariese, F. & Umapathy, S. Review of multidimensional data processing approaches for Raman and infrared spectroscopy. *EPJ Tech. Instrum.* **2:8,** 1–38 (2015).

7. Gok, S., Severcan, M., Goormaghtigh, E., Kandemir, I. & Severcan, F. Differentiation of Anatolian honey samples from different botanical origins by ATR-FTIR spectroscopy using multivariate analysis. *Food Chem.* **170,** 234–40 (2015).

8. Ballabio, D. & Todeschini, R. in *Infrared Spectroscopy for Food Quality Analysis and Control* (ed. Sun, D.-W.) 83–104 (Elsevier, 2009).

9. Stumpe, B., Engel, T., Steinweg, B. & Marschner, B. Application of PCA and SIMCA Statistical Analysis of FT-IR Spectra for the Classification and Identification of Different Slag Types with Environmental Origin. *Environ. Sci. Technol.* **46,** 3964–3972 (2012).

10. Grewal, M. K., Jaiswal, P. & Jha, S. N. Detection of poultry meat specific bacteria using FTIR spectroscopy and chemometrics. *J. Food Sci. Technol.* **52,** 3859–3869 (2014).

11. Duca, D. *et al.* Soft Independent Modelling of Class Analogy applied to infrared spectroscopy for rapid discrimination between hardwood and softwood. *Energy* **117,** 251–258 (2016).
